# Foliar water uptake as a source of hydrogen and oxygen in plant biomass

**DOI:** 10.1093/treephys/tpac055

**Published:** 2022-05-11

**Authors:** Akira Kagawa

**Affiliations:** Forestry and Forest Products Research Institute, Wood Anatomy and Quality Laboratory, Tsukuba 305-8687, Japan

**Keywords:** cellulose, pulse-labelling, stable isotopes, tree ring

## Abstract

Introductory biology lessons around the world typically teach that plants absorb water through their roots, but, unfortunately, absorption of water through leaves and subsequent transport and use of this water for biomass formation remains a field limited mostly to specialists. Recent studies have identified foliar water uptake as a significant net water source for terrestrial plants. The growing interest in the development of a new model that includes both foliar water uptake (in liquid form) and root water uptake to explain hydrogen and oxygen isotope ratios in leaf water and tree rings demands a method for distinguishing between these two water sources. Therefore, in this study, I have devised a new labelling method that utilizes two different water sources, one enriched in deuterium (HDO + D_2_O; δ*D* = 7.0 × 10 ^4^‰, δ^18^O = 4.1‰) and one enriched in oxygen-18 (H_2_^18^O; δ*D* = −85‰, δ^18^O = 1.1 × 10^4^‰), to simultaneously label both foliar-absorbed and root-absorbed water and quantify their relative contributions to plant biomass. Using this new method, I here present evidence that, in the case of well-watered *Cryptomeria japonica* D. Don, hydrogen and oxygen incorporated into new leaf cellulose in the rainy season derives mostly from foliar-absorbed water (69% from foliar-absorbed water and 31% from root-absorbed water), while that of new root cellulose derives mostly from root-absorbed water (20% from foliar-absorbed water and 80% from root-absorbed water), and new branch xylem is somewhere in between (55% from foliar-absorbed water and 45% from root-absorbed water). The dual-labelling method first implemented in this study enables separate and simultaneous labelling of foliar-absorbed and root-absorbed water and offers a new tool to study the uptake, transport and assimilation processes of these waters in terrestrial plants.

## Graphical Abstract

Suggested modification to photosynthesis diagrams. Before: a typical photosynthesis image shows only root water uptake as a source of hydrogen and oxygen in plant biomass. After: suggested modification shows foliar water uptake also as a source. This is the first study that quantifies relative contributions of hydrogen and oxygen from foliar-absorbed water and root-absorbed water to hydrogen and oxygen in plant biomass, and offers the first clear experimental evidence that a significant proportion of hydrogen and oxygen in the biomass (such as hydrogen and oxygen in cellulose) originates from foliar-absorbed water.

**Graphical Abstract:**
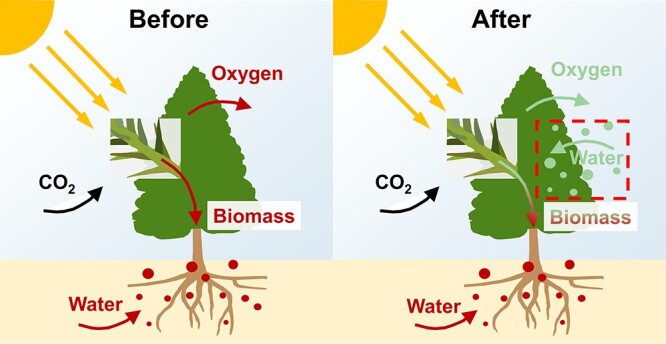

## Introduction

The concept that terrestrial plants absorb water with their roots and release water into the air through their leaves is common knowledge (McElrone et al. 2013) that has formed the basis for models for forest hydrology, atmospheric sciences and isotope dendroclimatology. However, in recent decades, several studies have pointed out the necessity of updating current models to explain various tree-physiological phenomena, such as tree water potential (Binks et al. 2019), isotope ratios of leaf water (Cernusak et al. 2018, Farquhar et al. 2021) and of tree rings (Kagawa and Battipaglia 2021), in response to growing evidence that plants can absorb some part of their water through their leaves (Burgess and Dawson 2004, Eller et al. 2013, Goldsmith et al. 2013, Gotsch et al. 2014, Dawson and Goldsmith 2018, Berry et al. 2019, Binks et al. 2020, Schreel and Steppe 2020).

The first indications that the general isotope model for explaining fractionations at the water evaporating surface (Craig and Gordon 1965) was too simplistic for explaining leaf water fractionations came in the 1980s from studies of fractionation of water in plant leaves (Leaney et al. 1985, Yakir and Deniro 1990). The ratio of heavy isotopes (δ*D* and δ^18^O) in leaf water tends to be elevated during transpiration because water molecules composed of heavy isotopes (HDO, D_2_O and $ {\rm H}_{2}^{18}{\rm O}$) evaporate more slowly from leaf surfaces than H_2_^16^O. Although the influence of this enrichment on a number of biogeochemical processes and the resulting ratios of oxygen isotopes in the atmosphere—including isotopes in carbon dioxide, a potential indicator of terrestrial gross primary productivity (Farquhar et al. 1993, Farquhar and Lloyd 1993, Cuntz et al. 2003, Welp et al. 2011) and isotopes in molecular oxygen—is now well accepted, the fate of enriched leaf water within plants is less understood. We also need to improve our understanding of oxygen isotope fractionation during photosynthesis, respiration and hydrologic processes (Dole effect) because it has been used as an indicator of the productivity of terrestrial plants (Dole et al. 1954, Luz et al. 1999, Hoffmann et al. 2004). Modifications of the ‘Craig–Gordon’ model (Craig and Gordon 1965, Dongmann et al. 1974) have tended to overestimate leaf water enrichment and have been further developed into two mechanistic models: the discrete-pool model and the Péclet model. The discrete-pool model views leaf water as two distinct fractions: evaporatively enriched water in the lamina and unenriched source water in the veins (Leaney et al. 1985, Yakir et al. 1989, 1990, 1994). The Péclet model takes into account both advection of vein water due to transpiration and backward diffusion of enriched water from evaporative sites, thus generating an isotopic gradient from veins to the evaporation site (Farquhar and Lloyd 1993, Farquhar and Gan 2003). However, despite huge efforts to further revise these models, they are still insufficient to describe observed isotopic distributions, and researchers have pointed out the necessity to improve our understanding of physiological controls over leaf water isotope enrichment and to develop new technologies to understand this phenomenon (Cernusak et al. 2016). Perhaps some discrepancies between current models and observations can be explained by foliar water uptake (net uptake of vapour and liquid water through the surface of wet leaves) in addition to root water uptake.

In the past, water in roots and stems was commonly assumed to have the same isotopic composition as the soil water (Gonfiantini et al. 1965, Wershaw et al. 1966, Dawson and Ehleringer 1991). However, in light of current research showing that foliar water uptake provides plants with a significant secondary water source (Eller et al. 2013, Goldsmith et al. 2013, Gotsch et al. 2014, Dawson and Goldsmith 2018, Berry et al. 2019, Binks et al. 2019, Schreel and Steppe 2020), we should expect isotopic differences between precipitation and soil water (Treydte et al. 2014) to be reflected in different parts of plants (Zhao et al. 2016, De Deurwaerder et al. 2020). When water vapour concentrations in leaf intercellular spaces are higher than those in the air, foliar vapour exchange with unsaturated air cannot cause reverse advectional flow of water in veins (Farquhar and Cernusak 2005, Vesala et al. 2017, Cernusak et al. 2018). However, net foliar uptake of liquid water can cause a significant amount of reverse flow (Eller et al. 2013, Cassana et al. 2016, Binks et al. 2019). Since net foliar water uptake—which involves both foliar vapour exchange and net foliar uptake of liquid water—is associated with reversals in sap flow, we might hypothesize the existence of a gradient in the ratio of foliar-absorbed water to root-absorbed water (FAW/RAW), with the ratio decreasing across tissue water from leaves to roots, and FAW even reaching the roots in some cases (Eller et al. 2013, Cassana et al. 2016), and this gradient may be then reflected in tree biomass in a significant way because tree growth is enhanced during and immediately after a rain event (Zweifel et al. 2021). Adequate assessment of this possibility requires investigating the contribution of FAW and RAW to the isotopic gradient along the stem as well as in the roots and leaves, and in both tissue water and biomass.

Understanding how leaf water and cambial water isotope signals are then incorporated into tree-ring cellulose is important for applications such as reconstruction of paleoclimate and analysis involving plant responses to environmental changes (Gessler et al. 2014). In East Asia, archaeological research has come to rely more and more on tree-ring oxygen isotopes for isotopic cross-dating (Roden 2008, Loader et al. 2019, Choi et al. 2020, Nakatsuka et al. 2020) and provenancing of timber (Kagawa and Leavitt 2010). The precision of these methods depends on within-site coherence and mechanistic understanding of tree-ring δ^18^O. The isotopic mass balance model of leaf water predicted that significant amount of atmospheric vapour can enter the leaf via bidirectional vapour exchange (Farquhar and Cernusak 2005, Kim and Lee 2011, Goldsmith et al. 2017, Farquhar et al. 2021), and incorporation of the vapour into biomass through stomata is assumed by the model (Roden et al. 2000). However, relative contributions of foliar-absorbed vapour and root-absorbed water to hydrogen and oxygen in plant biomass remained unknown. The idea that liquid FAW, in addition to atmospheric vapour, may be incorporated into biomass of epiphytes has been explored previously (Helliker 2011, 2014); however, as far as other types of plants that rely on RAW are concerned, the topic received serious attention only in recent years. For example, Studer et al. (2015) and Lehmann et al. (2018, 2019) used hydrogen and oxygen isotope tracer in vapour or mist form to demonstrate how FAW can be assimilated into organic matter, but, until recently, relative contributions of FAW and RAW assimilation into tree rings have remained unknown (Kagawa 2020, Kagawa and Battipaglia 2021). It is therefore vital that we investigate similar possibilities for tree-ring cellulose in climates with a wet growing season. In the past, dual-isotope (HDO + D_2_O and $ {\rm H}_{2}^{18}{\rm O}$) labelling has been used to study the depth of soil water uptake by trees (Stahl et al. 2013). In this study, I applied this dual-isotope labelling method to separately and simultaneously label FAW and RAW. To the best of my knowledge, this is the first time dual-isotope labelling with two isotopically contrasting water sources has been applied (i) to study uptake and transport of FAW and RAW within a whole tree and (ii) to assess the relative contributions of FAW and RAW to organic matter of trees.

According to Köppen’s climate classification, the Japanese mainland is characterized by a ‘humid subtropical climate’ (Kottek et al. 2006), in which the summer monsoon brings a marked rainy season (commonly called the ‘plum rain’, 雨, in countries in this region). During the rainy season, overcast conditions with photosynthetic photon flux density (PPFD) of less than 1000 μmol m^−2^ s^−1^ are much more frequent than sunny conditions with PPFD around 2000 μmol m^−2^ s^−1^ (Miyashita et al. 2012, see Figure S1 available as Supplementary data at *Tree Physiology* Online). Canopy leaves stay wet during and immediately after a rain event, and also when dews are formed (Klemm et al. 2002, Binks et al. 2020). In Tsukuba, where this study was conducted, an average rainy season lasts from 8 June to 21 July (Japan Meteorological Agency, https://www.jma.go.jp/), which corresponds to the latter half of the earlywood formation period and includes the summer solstice when radial tree growth is at a maximum (Kagawa et al. 2005, Rossi et al. 2006).

With the requirement for leaf wetness and high humidity during the growing season met, we might suspect that foliar water uptake will have an especially strong impact on hydrogen and oxygen isotope ratios in trees in Japan and surrounding regions. This suspicion grows if we consider that, according to statistical isotope dendroclimatology data from various parts of the world, tree-ring δ^18^O series from regions with relatively wet growing seasons tend to show both higher correlation to early-summer humidity and precipitation, and higher within-site coherence. For example, oxygen isotope ratios of tree rings from Japan and Korea—including tree-ring δ^18^O series for Japanese cedar (*Cryptomeria japonica*) with some of the highest known degrees of coherences in the world—show very high correlations to precipitation and humidity during the rainy season (Nakatsuka et al. 2004, 2020, Li et al. 2015, Seo et al. 2019). In the Pacific Northwest, which has a similarly wet growing season, foliar water uptake has been directly observed in coastal redwoods (*Sequoia sempervirens*) with up to 6% of leaf water originating from the previous night’s fog (Burgess and Dawson 2004, Limm et al. 2009), and tree rings of coastal redwood also show strong coherence at both inter-annual and intra-annual levels (Roden 2008, Roden et al. 2009, 2011). In contrast, in regions with a Mediterranean climate, for example, precipitation and humidity reach a minimum in the middle of the growing season (Kottek et al. 2006), and tree-ring δ^18^O shows weaker coherence (Konter et al. 2014, Shestakova et al. 2014).

This is understandable if we consider that isotope ratios of rainwater and atmospheric vapour are strongly coherent from catchment to regional scales, especially in humid subtropical climate (Miyake et al. 1968, Ingraham 1998, Treydte et al. 2014, Baker et al. 2015, Fan et al. 2021), whereas isotope ratios of soil water are less coherent because of the heterogeneous nature of soil (Dawson 1993, Tsujimura and Tanaka 1998, Treydte et al. 2014). Another cause of the low coherence of Mediterranean tree-ring δ^18^O is heterogeneous δ^18^O of precipitation in the region during summer. Precipitation in this region is influenced more by recycled continental moisture and influenced less by oceanic moisture compared with that in Asian monsoon region (Fan et al. 2021, Moreno et al. 2021), where the former moisture is brought from local convective clouds and the latter from large-scale precipitation fronts.

Although foliar water uptake may be an important water source for Mediterranean trees during dry summer (Fernández et al. 2014), growing-season humidity is lower in Mediterranean climate and thus the relative contribution of FAW (both in liquid and vapour phase) to hydrogen and oxygen of plant biomass may be smaller compared with trees in Asian monsoon regions. We can therefore hypothesize that tree-ring hydrogen and oxygen isotopic series with higher contributions of FAW may show larger coherence across a study site.

**Figure 1.**
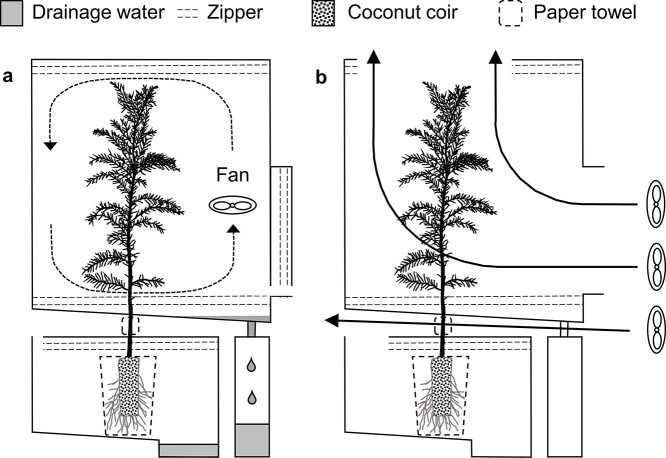
Chambers for dual-isotope pulse-labelling. (a) To label O/H trees (trees A, B and C), needles were sprayed with heavy oxygen water ($ {\rm H}_{2}^{18}{\rm O}$) and soil was watered with heavy hydrogen water (HDO + D_2_O). (b) To label H/O trees (trees D, E and F), needles were sprayed with heavy hydrogen water and soil was watered with heavy oxygen water. After labelling, chambers were exposed to gentle wind so that vapours potentially leaking from chambers would not enter other chambers.

Our current mechanistic understanding of the assimilation of FAW into tree rings has been limited by the lack of effective tools to study this phenomenon (Berry et al. 2019). Authors including myself have suggested that use of artificially enriched tracers in the forms of ^13^CO_2_, HDO + D_2_O and $ {\rm H}_{2}^{18}{\rm O}$ instead of natural isotope tracers may provide a clearer picture of the physiological processes between photosynthetic incorporation of isotopes and their deposition into tree rings (Roden and Ehleringer 1999, Helle and Panferov 2004, Kagawa et al. 2005, 2006a, Kagawa and Battipaglia 2021). I have therefore devised a dual-isotope labelling method utilizing HDO + D_2_O and $ {\rm H}_{2}^{18}{\rm O}$ to trace absorption, transport and assimilation of water at the whole-plant level (Figure 1, Kagawa 2020), which are particularly appealing because hydrogen and oxygen isotopes behave similarly in the environment (Craig and Gordon 1965) and tree-ring isotope ratios of hydrogen and oxygen are explained by the same mechanistic model with different fractionation factors (Roden et al. 2000, Gessler et al. 2014). The dual-isotope labelling method allows separate and simultaneous labelling of FAW and RAW with two isotopically contrasting water sources.

I used the dual labelling method to test my hypothesis that a significant amount of FAW can be assimilated into plant biomass, and the following three questions were addressed in this paper: (i) what is the rate of foliar water uptake and when does it reach equilibrium; (ii) does the FAW/RAW ratio differ across needles, phloem + epidermis, xylem and roots; and (iii) what percentage of hydrogen and oxygen in cellulose and dry organic matter originates from FAW? In order to answer these questions, Japanese cedar (*C. japonica*) was chosen as my subject. Japanese cedar is one of the most frequently studied trees in (isotope) dendroclimatology research in Japan while their North American siblings in the sequoia genus (family Cupressaceae) are similarly well known in research. For example, redwood (*Sequoia sempervirens*) is known as the tallest tree species on Earth, and even with ample soil moisture, increasing leaf water stress due to gravity and path length resistance is likely to limit its height (Koch et al. 2004), unless foliar water uptake can provide a second source of water (Burgess and Dawson 2004). Likewise, foliar water uptake may play an important role for Japanese cedar, which achieves the tallest height among Japanese tree species.

Foliar water uptake is also one of the most important mechanisms driving active canopy interception (Breshears et al. 2008), and I hypothesize that maximal transport of FAW towards roots occurs at the beginning of a rain event, as illustrated by Figure 1c in Goldsmith (2013) because water potential difference between leaves and roots should be large when leaves start getting wet and roots are still dry (Eller et al. 2013, Cassana et al. 2016). This situation is likely to happen especially at a mature forest with closed canopy.

In this study, one ‘O/H’ group of saplings were pulse-labelled by spraying needles with heavy oxygen water and watering roots with heavy hydrogen water, and a second ‘H/O’ group of saplings by spraying needles with heavy hydrogen water and watering roots with heavy oxygen water (Figure 1). By analysing the subsequent hydrogen and oxygen isotopic signals of the tissue water and biomass in each organ, I herein attempt to shed light on the absorption, transport and assimilation of FAW and root-absorbed water into the structural components of trees.

## Materials and methods

### Sample trees and labelling experiment

For this experiment, eight 2-year-old saplings of Japanese cedar were used, grown at the Forestry and Forest Products Research Institute in Tsukuba, Japan. Each sapling grew in a pot (inner diameter 45 mm, depth 135 mm), filled with coconut coir and fertilized with slow-release nitrogen, phosphorus and potassium. The pots were moved to a nearby greenhouse for acclimatization 1 month before the labelling experiment. During this period, they were watered every 2–3 days to field capacity with tap water. The greenhouse was well ventilated with open side windows and equipped with a glass roof to shelter the saplings from rain. Average sapling height was 45 ± 14 cm at the time of labelling.

The saplings were divided into three groups: the O/H group consisting of trees A, B and C would have their aboveground parts supplied with heavy oxygen water and their belowground parts supplied with heavy hydrogen water. The H/O group consisting of trees D, E and F would have their aboveground parts supplied with heavy hydrogen water and their belowground parts supplied with heavy oxygen water. The control group consisting of two trees G and H would be supplied only tap water.

On 16 July at 15:00h, separate chambers constructed from transparent, resealable plastic bags were installed around the aboveground parts and belowground parts of each sapling, as shown in Figure 1a. The seal around the stem was reinforced where it exits the aboveground chambers with Gaffer tape, leaving a 5-cm gap between chambers, where each stem was gently wrapped in paper towel. The bottom hem of each aboveground chamber was turned up towards the inside to create a gutter leading condensation towards a drainage bottle. Soil drained to a bottom corner in the ground chambers. Throughout the experiment, the air inside each aboveground chamber was gently circulated by an electric fan. The two control saplings (trees G and H) were placed immediately downwind of the experimental saplings to detect potential interference of heavy-water vapour escaping the chambers, which may lead to cross-contamination of the pulse-labelled trees. To prevent condensation, chamber seals were left open until after the first labelling procedure.

On 16 July at 18:00h, around sunset, a sprayer was used to wet the surfaces of the upper needles of tree A with heavy oxygen water (2.3 atom% ^18^O). After closing the top seal, the side seal was opened and the middle and lower needles were similarly wetted, injecting a total of 15.4 ml of heavy oxygen water into the aboveground chamber. Then the side seal was closed, leaving open a small vent just large enough for atmospheric gases to enter the chamber and maintain a CO_2_ concentration above 200 p.p.m. Next the seal of the ground chamber was opened. A pipette was used to uniformly wet the upper surface of the soil with 12 ml of heavy hydrogen water (1.1 atom% D), then closed the seal, except for a CO_2_ vent (Figure 1a). The same procedure was repeated on trees B and C, and the whole process for the three saplings in the H/O group were repeated, but with the heavy water supplies swapped for the aboveground and belowground parts. All of this was completed within 30 min of 18:00h. On 17 and 18 July at 06:00, 12:00 and 18:00h, this labelling process was repeated for both groups of trees. In total, the aboveground parts of each tree received 108 ml of heavy water and the belowground parts of each tree received 84 ml of heavy water. These values were chosen in consideration of reports that, under the typical growing conditions for the trees, 22% of rainfall does not reach the ground due to interception mechanisms including foliar water uptake (Murakami 2006, Breshears et al. 2008).

After the last administration of heavy water, the aboveground chambers were kept sealed except for CO_2_ vents for 24 h to emulate high-humidity conditions for 1 day following a rain event. On 19 July at 18:00h, the drainage water was collected and the chambers were opened to outside ventilation (Figure 1b) until 20 July at 06:00h, when the chambers were completely removed. The saplings were weighed on 20, 21, 22, 24 and 26 July to measure transpiration (Seiler and Johnson 1988), and they were watered with tap water (15.4 ml to aboveground parts and 12 ml to belowground three times a day) through 18:00h on 26 July, at which point saplings were transplanted to 4-l pots to accommodate growing root systems and the pots were watered to field capacity three times. From this date onward, saplings were watered with tap water every 2–3 days to field capacity during the growing season (until October 2019), including wetting the needles and watering the soil, then every 7 days during the dormant period (after November 2019). Moderate shoot and root growth were observed between August and October 2019. Temperature and CO_2_ concentration were monitored inside the chambers from 16 to 19 July, and outside the chambers from 15 July to 7 August.

**Figure 2.**
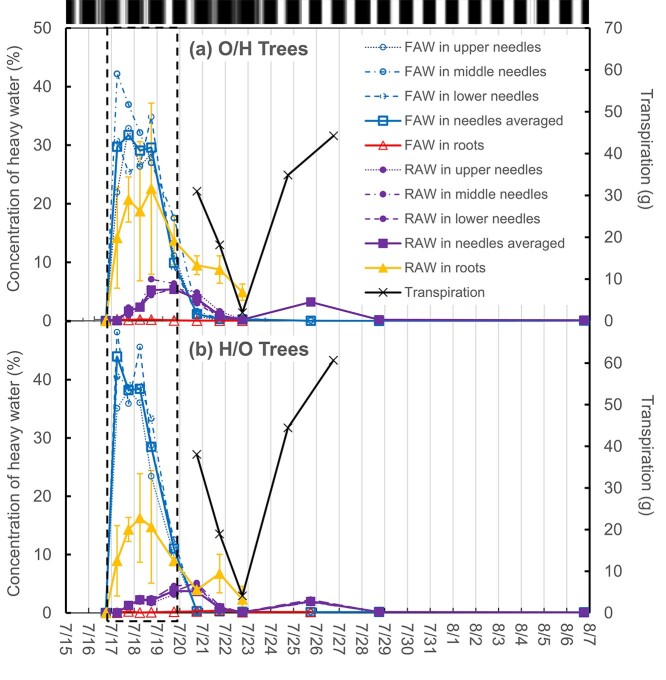
Absorption and transport of heavy FAW and heavy RAW within trees. (a) Averaged concentrations of heavy water in needles and roots of three O/H trees. Needles and roots of these trees were exposed to heavy oxygen water and heavy hydrogen water, respectively. (b) Averaged concentrations of heavy water in needles and roots of three H/O trees. Needles and roots of these trees were exposed to heavy hydrogen water and heavy oxygen water, respectively. Labelling with heavy water was conducted from 16 July at 18:00h to 19 July at 18:00h (dashed box). RAW in roots was more erratic than needles and therefore indicated with error bars, representing standard deviation. For reference, the diurnal patterns of PPFD in Figure S1 available as Supplementary data at *Tree Physiology* Online are illustrated by the grey-coloured map at the top of the figure.

Needles were sampled from upper, middle and lower branches, and new roots (easily distinguishable from older roots by their white appearance) periodically from 16 July at 17:30h (before labelling) through 25 July (6 days after the last labelling) and needles were sampled again on 28 July and 6 August. Samples were limited to volumes the saplings could tolerate without damage and sampling frequency was reduced as necessary, as shown in Figure 2 and Figure S2 available as Supplementary data at *Tree Physiology* Online. When samples were wet, the wet surfaces were wiped dry, then all samples were sealed in air-tight vials and put immediately into a freezer (−14 °C) located at the greenhouse before transfer to a larger freezer (−30 °C) closer to our microbalance.

The following January, roots and current-year branches were sampled for α-cellulose extraction. The branch was sampled halfway between the positions where upper and middle needles were sampled. To minimize damage to the root system and keep the trees healthy for later observation, I used roots formed within the soil added around the original coconut coir. α-Cellulose was extracted from these samples and also from needles and roots sampled previously on 25 July according to the methods of Laumer et al. (2009) and Kagawa et al. (2015). Needles, phloem and epidermis were removed from current-year branches. Needles were dried, ground in a steel ball mill (ICL, Wig-L-Bug Model 30) and extracted with 3:1 chloroform/ethanol. Phloem + epidermis and xylem samples were sliced into thin slivers using a razor. Chloroform-extracted needles, slivered phloem + epidermis and slivered xylem samples were next extracted with ethanol (60 °C, 48 h) and reverse osmosis water (100 °C, 48 h), acidified sodium chlorite (70 °C, 12 h) and NaOH (80 °C, 12 h). Finally, the resulting α-cellulose was rinsed, ultrasonically homogenized and freeze-dried (Laumer et al. 2009).

### Stable isotope analysis and calculation of isotope data

For this analysis, in addition to measurements of the control trees G and H, the hydrogen and oxygen isotope ratios were measured in the following samples: upper, middle and lower needles and fine roots (fresh samples) of all O/H and H/O saplings (trees A, B, C, D, E and F); the organic matter of upper, middle and lower needles, and fine roots (dried samples) of all six saplings; the α-cellulose of needles, phloem + epidermis, xylem, and roots of all six saplings; the drain water from the aboveground and ground chambers of all six saplings; and the heavy hydrogen water, heavy oxygen water and local (Tsukuba) tap water.

Before the analysis of each plant sample, the vial was removed from the freezer and it was allowed to approach room temperature for a few minutes to prevent condensation. Then, working quickly to minimize evaporative losses, an approximately 1-mm segment was removed from the cut surfaces of each needle or piece of fine roots to prevent potential contamination from heavy water absorbed from the cut surfaces, then the rest of each piece was divided into three segments of roughly equal length and the central segment was placed in preweighed smooth-walled tin capsules designed for liquids. In order to examine if there was any contamination of heavy water absorbed from cut surfaces, I selected some of the needles, and subdivided each needle into 4–16 pieces and packed each subdivision into a tin capsule. Later analysis showed that there was no sign of heavy water contamination from the cut surfaces (data not shown). The lip of each capsule was folded over and the capsule was pressed closed three times to make the seal airtight. These capsules were weighed, and placed in the carousel of a Zero Blank autosampler (Costech), and the rotary vacuum pump connected to the autosampler was run for more than 1 h before the capsules were weighed again to check for leaks. Meanwhile, the remaining outer pairs of needle and root segments were placed in preweighed tin capsules designed for solids, weighed and dried in a vacuum oven at 70 °C for more than 24 h, and they were weighed again to calculate the water content of needle and root segments. Samples of heavy water, drainage water from the chamber vials, and reference water standards were also weighed into smooth-walled tin capsules. For reference standards, artificially enriched and natural water (IAEA-607, IAEA-608, IAEA-609, International Atomic Energy Agency and our internal standard) and natural cellulose (IAEA-C3 cellulose, Merck cellulose) with known isotope ratios measured against the Vienna Standard Mean Ocean Water (VSMOW) standard (detailed in the next section) were used. To increase precision, five or more capsules were prepared from each sample of water and cellulose (Olsen  et al. 2006), except when limited by sample volume, and two to five capsules were prepared from each needle and root sample, depending on the amount of sample available.

To analyse the hydrogen and oxygen isotope ratios of solid, liquid and wet tissue (= solid + liquid) samples, a high-temperature pyrolysis system (Hekatech, HTO) was used, which was coupled via thermal conductivity detector to a mass spectrometer (Thermo-Finnigan, Delta V Advantage) with a longer gas chromatography column (molecular sieve 5A, 2 m, i.d. 5 mm) to achieve clear separation of hydrogen, nitrogen and carbon monoxide (Gehre and Strauch 2003). To minimize background moisture and nitrogen gas from the samples, the three-way valve connected to the carousel chamber of the autosampler was opened to the vacuum pump for 30 s, then the valve was switched to the helium gas supply for 30 s, and this procedure was repeated 10 or more times until the background levels (mass = 28, 29, 30) stabilized, before initiating analysis (Kagawa et al. 2015). To minimize the impact of sample-to-sample memory, plant and water samples with similar isotope ratios were grouped together in the analytical sequence. The pyrolysis tube was replaced as necessary due to tin accumulation, typically after processing 50 samples in smooth-walled tin capsules.

### Calculation of isotope data

The calculations of excess D and ^18^O in the labelled samples were based on published methodologies for calculating excess ^13^C of ^13^CO_2_ pulse-labelled samples as follows (Boutton 1991, Kagawa et al. 2006*b*). For each sample, δ*D* and δ^18^O (*δ*) were first converted to absolute isotope ratio (*R*_sample_)

(1) \begin{equation*} {}^n{R}_{\mathrm{sample}}=\left({}^n\delta /1000+1\right)\kern0.33em \cdot{\kern0.33em }^n{R}_{\mathrm{std}}, \end{equation*}

where *n* is the mass number of the heavy hydrogen or oxygen isotopes and *R*_std_ is the absolute isotope ratios of VSMOW: ^2^*R*_std_ = 0.00015576, ^17^*R*_std_ = 0.000379 and ^18^*R*_std_ = 0.0020052; De Wit et al. (1980). The D and ^18^O abundance ratios (*A*) were calculated as follows:

(2) \begin{equation*} {}^2A={\kern0.33em }^2R/\left({}^2R+1\right) \end{equation*}

and

(3) \begin{equation*} {}^{18}A={\kern0.33em }^{18}R/\left({}^{18}R+{\kern0.33em }^{17}R+1\right). \end{equation*}

Then excess D and ^18^O abundance (Δ^n^*A*) was calculated as the deviation from natural isotopic values

(4) \begin{equation*} {\varDelta}^{\mathrm{n}}A={\kern0.33em }^{\mathrm{n}}{A}_{\mathrm{sample}}-{\kern0.33em }^{\mathrm{n}}{A}_{\mathrm{BL}}, \end{equation*}

where *A*_BL_ and *A*_sample_ are abundance ratios of the samples taken before and after labelling, respectively. Here is a formula describing the isotopic mass balance

(5) \begin{equation*} {\varDelta}^{\mathrm{n}}{A}_{\mathrm{F}}=x\cdot{\varDelta}^{\mathrm{n}}{A}_{\mathrm{TW}}+\left(1-x\right)\cdot{\varDelta}^{\mathrm{n}}{A}_{\mathrm{D}}, \end{equation*}

where Δ*A*_F_ and Δ*A*_D_ are the excess abundances of fresh and dry samples of needles or roots, and *x* refers to the portion of hydrogen or oxygen contributed by tissue water (leaf water or root water) to the total hydrogen or oxygen of the fresh sample, calculated from the water and elemental content of fresh and dry samples (for details, see Gan et al. 2004). From this formula, excess abundance of tissue water (Δ*A*_TW_) was calculated as

(6) \begin{equation*} {\varDelta}^{\mathrm{n}}{A}_{\mathrm{TW}}=\left({\varDelta}^{\mathrm{n}}{A}_{\mathrm{F}}-\left(1-x\right)\cdot{\varDelta}^{\mathrm{n}}{A}_{\mathrm{D}}\right)/x. \end{equation*}

Then, τ*_H_* and τ*_O_* were introduced, the percentages of hydrogen and oxygen, respectively, in samples replaced by atoms from the heavy water and calculated as

(7) \begin{equation*} {\tau}_{\mathrm{H}}={\varDelta}^2{A}_{\mathrm{sample}}/{\varDelta}^2{A}_{\mathrm{H}\mathrm{W}} \end{equation*}

and

(8) \begin{equation*} {\tau}_{\mathrm{O}}={\varDelta}^{18}{A}_{\mathrm{sample}}/{\varDelta}^{18}{A}_{\mathrm{HW}}, \end{equation*}

where Δ^n^*A*_HW_ is the excess abundance of heavy water compared with local tap water.

Hydrogen and oxygen isotope fractionations show similar patterns within hydrological cycles along the meteoric water line (Dansgaard 1964), but they show difference within biological systems, at the leaf water level (Yakir et al. 1990), at the cambium (Yakir and DeNiro 1990) and at tree rings (Nabeshima et al. 2018, Nakatsuka et al. 2020). Such deviations in isotopic fractionation between hydrogen and oxygen seem to be related to metabolic activities (Lehmann et al. 2020, Nakatsuka et al. 2020), and one possible cause for such deviations is respiration because oxygen is eliminated by both carbon dioxide and water from living organisms, and hydrogen is eliminated only by water. Although heavy hydrogen water and heavy oxygen water show similar exponential decrease after their incorporation into an organism, concentrations of heavy hydrogen and heavy oxygen water show slight deviation over time. The deviations between oxygen and hydrogen isotopes are used to assess amount of respired CO_2_ in humans and animals (Schoeller and Van Santen 1982, Speakman 1997), and a similar phenomenon might be happening in plants (Kagawa and Battipaglia 2021). To offset the effect of any chemical difference between hydrogen and oxygen, and to calculate the relative contribution of FAW and RAW assimilated into biomass (I focus on cellulose in this article) by weight, τ_Cell_ was calculated, the weight percentage of hydrogen + oxygen in cellulose replaced by heavy water. Within each cellulose unit (C_6_H_10_O_5_), there are seven non-exchangeable carbon-bound hydrogen atoms, and three exchangeable hydrogen atoms in OH bonds. Since my cellulose samples were not nitrified, hydrogen atoms in the three OH bonds of each cellulose unit were assumed to be replaced by natural hydrogen atoms while boiling in natural water overnight, because OD (oxygen-deuterium) moieties of cellulose can be easily rehydrogenated by soaking cellulose in hot water (Horikawa and Sugiyama 2008), and therefore multiplied cellulose τ_H_ by a factor of 1.43 (=10/7).

(9) \begin{equation*} {\tau}_{\mathrm{Cell}}=\left(1.43\cdot 10\cdot{\tau}_{\mathrm{H}}+80\cdot{\tau}_{\mathrm{O}}\right)/\left(10+80\right), \end{equation*}

where 10 and 80 are molecular weights of hydrogen and oxygen in each cellulose unit. To check for differences between O/H and H/O trees, the corresponding τ of α-cellulose were compared across four different parts (needles, phloem + epidermis, xylem and roots), using an *F*-test (two-way ANOVA with three replications, MS Excel 2016).

Because hydrogen and oxygen show similar biochemical behaviours within the various parts of trees, hydrogen and oxygen isotope ratios of organic matter of various tissues (δ*D_t_* and δ^18^O*_t_*)—including needles, phloem, epidermis, xylem and roots—can be explained by the same mechanistic model as follows (Roden et al. 2000):

(10) \begin{equation*} \delta{D}_{\mathrm{t}}={q}_{\mathrm{ex}}\cdot \left(\delta{D}_{\mathrm{wt}}+{\varepsilon}_{\mathrm{HH}}\right)+\left(1-{q}_{\mathrm{ex}}\right)\cdot \left(\delta{D}_{\mathrm{wl}}+{\varepsilon}_{\mathrm{HA}}\right) \end{equation*}

(11) \begin{equation*} {\delta}^{18}{O}_t={p}_{\mathrm{ex}}\cdot \left({\delta}^{18}{O}_{\mathrm{wt}}+{\varepsilon}_{\mathrm{O}}\right)+\left(1-{p}_{\mathrm{ex}}\right)\cdot \left({\delta}^{18}{O}_{\mathrm{wl}}+{\varepsilon}_{\mathrm{O}}\right), \end{equation*}

where the subscripts wt refers to local tissue water at non-green parts of the tree and wl refers to leaf water at the chloroplast. The hydrogen and oxygen available for exchange with local tissue water are represented by *q_ex_* and *p_ex_*, respectively. The hydrogen isotope fractionation factors associated with heterotrophic and autotrophic metabolism are expressed as ε_HH_ (= +158‰) and ε_HA_ (= −171‰), respectively. For oxygen, there is no need to distinguish between the two, hence both are expressed as ε*_O_* (= +27‰) (Roden et al. 2000).

We are specifically interested in δ*D_t_* and δ^18^O*_t_* of new tissues (formed during and immediately after the pulse-labelling). Δ*D_t_* and Δ^18^O*_t_* can be related to τ*_H_* and τ*_O_* of whole tissue sample (τ_Ht_ and τ_Ot_), as follows:

(12) \begin{equation*} \Delta{D}_t={\tau}_{\mathrm{Ht}}/r \end{equation*}

(13) \begin{equation*} {\Delta}^{18}{O}_t={\tau}_{\mathrm{Ot}}/r, \end{equation*}

where τ_Ht_ and τ_Ot_ can be calculated from measured isotope ratios by mass spectrometer, and *r* is proportion of H and O in new (pulse-labelled) tissue to those of whole sampled tissue (which contains old tissue with natural hydrogen and oxygen isotope ratios).

### Calculation of isotope exchange

Compared with oxygen, less research is available concerning hydrogen isotope exchange (Yakir and DeNiro 1990, Terwilliger and DeNiro 1995, Waterhouse et al. 2002), simply because it is more difficult to analyse natural-abundance isotope ratios of carbon-bound hydrogen in plant biomass. As I later discuss in Assimilation of FAW and RAW into cellulose, similarity of heavy water distributions between the O/H and H/O trees (Figure 2, Figure S2 available as Supplementary data at *Tree Physiology* Online) supported the assumption that *q*_ex_ ≈ *p*_ex_ in our approach. I therefore introduced *pq*_ex_, the proportion of exchange that is common to both hydrogen and oxygen in order to simplify calculations, and assumed that *q*_ex_ ≈ *p*_ex_ ≈ *pq*_ex_, an assumption supported by previous studies (Yakir and DeNiro 1990, Roden and Ehleringer 1999, Roden et al. 2000). Considering that the excess δ*D* and δ^18^O of the heavy water was more than 400 times larger than the absolute values of ε_HH,_ ε_HA_ and ε_O_, contributions from ε_HH_, ε_HA_ and ε_O_ were deemed to be negligible compared with δD_wt_, δD_wl_, δ^18^O_wt_ and δ^18^O_wl_. Therefore, to estimate fractions of hydrogen and oxygen exchange with the water at the cambium, the following formulae were used

(14) \begin{equation*} \Delta{D}_t=p{q}_{\mathrm{ex}}\cdot \Delta{D}_{\mathrm{wt}}+\left(1-p{q}_{\mathrm{ex}}\right)\cdot \Delta{D}_{\mathrm{wl}} \end{equation*}

(15) \begin{equation*} {\Delta}^{18}{O}_t=p{q}_{\mathrm{ex}}\cdot{\Delta}^{18}{O}_{\mathrm{wt}}+\left(1-p{q}_{\mathrm{ex}}\right)\cdot{\Delta}^{18}{O}_{\mathrm{wl}}, \end{equation*}

where Δ*D*_wt_ and Δ^18^O_wt_ are time-integrated τ*_H_* and τ*_O_* of tissue water and Δ*D*_wl_ and Δ^18^O_wl_ are time-integrated τ*_H_* and τ*_O_* of leaf water. Δ*D*_wl_, Δ^18^O_wl_, Δ*D*_wt_ and Δ^18^O_wt_ can be calculated as time-integrals of τ*_H_* and τ*_O_* values of leaf water and root water from data indicated in Figure 2 (see Table 4 for details). By solving Eqs (14) and (15) simultaneously for *r* and *pq*_ex_, fractions of hydrogen and oxygen exchange can be calculated from measured isotopic data as follows:

(16) \begin{align*} {{pq}}_{{ex}}=&\left[{\tau}_{\mathrm{Ht}}\cdot{\varDelta}^{18}{O}_{\mathrm{wl}}-{\tau}_{\mathrm{Ot}}\cdot \varDelta{D}_{\mathrm{wl}}\right]/\left[{\tau}_{\mathrm{Ot}}\cdot \left(\varDelta{D}_{\mathrm{wt}}-\varDelta{D}_{\mathrm{wl}}\right)\right.\nonumber\\ &+\left.{\tau}_{\mathrm{Ht}}\cdot \left({\varDelta}^{18}{O}_{\mathrm{wl}}-{\varDelta}^{18}{O}_{\mathrm{wt}}\right)\right]. \end{align*}

Roots were examined in particular because roots were the only non-green tissue that could be repeatedly sampled without damaging the trees.

## Results

### Preliminaries

In Tsukuba, the rainy season of 2019 lasted from 7 June to 24 July (Japan Meteorological Agency, https://www.jma.go.jp/), which puts our pulse-labelling period (16–19 July) near the end of the rainy season. The weather during the pulse-labelling period (dotted square in Figure S1 available as Supplementary data at *Tree Physiology* Online) was cloudy, emulating environmental conditions where foliar water uptake naturally happens. The 4 days following the pulse-labelling had low solar radiation with occasional rain and 24 July onwards saw a series of sunny days with temperature exceeding 30 °C. Transpiration was proportional to PPFD, and, when averaged among six trees, they transpired an average of 53 g each on a sunny day and between 3 and 35 g each on rainy/cloudy days (Table S1 available as Supplementary data at *Tree Physiology* Online).

The preliminary analysis of heavy water gave the following results, which were used in subsequent sample analysis: heavy hydrogen water had ^18^O concentration within the natural range and deuterium concentration of 1.1%, which is 72 times higher than in natural water. Heavy oxygen water had a deuterium concentration within the natural range and ^18^O concentration of 2.3%, 12 times higher than in natural water (Table 1). Based on these values, a 960‰ increase in excess δ*D* translates to a 1% increase in τ*_H_*, and a 140‰ increase in excess δ^18^O translates a 1% increase in τ*_O_* (Figure 2, Figure S2 available as Supplementary data at *Tree Physiology* Online).

**Table 1:** Isotope ratios and atom abundances in heavy water and tap water

	Heavy hydrogen water	Heavy oxygen water	City of Tsukuba tap water
δ^2^H (‰, VSMOW)	69,999 ± 1,078	−85 ± 1	−28.1 ± 0.7
^2^H (Δ^2^*A*_HW_) (atom%)	1.0938	0.0143	0.0151
δ^18^O (‰, VMOW)	4.1 ± 0.7	10,845 ± 110	−5.4 ± 0.2
^18^O (Δ^18^*A*_HW_) (atom%)	0.201	2.319	0.1994

Heavy hydrogen water had an oxygen-18 concentration within natural range and deuterium concentration 72 times higher than natural water. Heavy oxygen water had a deuterium concentration within natural range and oxygen-18 concentration 12 times higher than natural water.

The analysis of needles and roots sampled from the control trees (trees G and H) before and after pulse-labelling experiment showed no significant increase for δ^18^O in needle and δ^18^O and δ*D* in root water (one-tailed *t*-test, *P* > 0.05). There was a significant increase in δ*D* of needle water (+24.4‰, *P* = 3.1 × 10^−5^), but still within the natural variation (±26‰) according to Cernusak et al. (2016). I therefore concluded that any cross-chamber contamination was within acceptable limits.

### Isotopic signals of FAW and RAW in tissue water

The heavy water concentrations in needles and roots showed similar trends in both O/H saplings (Figure 2a, Figure S2a–c available as Supplementary data at *Tree Physiology* Online) and H/O saplings (Figure 2b, Figure S2d–f available as Supplementary data at *Tree Physiology* Online). Needles sampled during the labelling period showed the highest water content at the upper part (62.2 ± 1.2%, *n* = 10), followed by middle (57.0 ± 5.8%, *n* = 10) and lower parts (51.2 ± 3.0%, *n* = 10), as previously observed in mature trees of this species (Azuma et al. 2016, Azuma et al. 2020), however little among upper, middle and lower needles (Figure S2 available as Supplementary data at *Tree Physiology* Online). Almost half of needle water was replaced by heavy FAW within 24 h of heavy water exposure. After the third or fourth sprays of heavy water (18 July), heavy FAW in needles started decreasing, and within 72 h of opening each chamber on 19 July, 18:00h (Figure 1b), the heavy FAW signal in needles was completely lost (22 July, 18:00h).

Heavy RAW was first detected in needles 24 h after the first heavy water administration (17 July, 18:00h) and reached a maximum around the end of the labelling period (19 July, 18:00h to 20 July, 18:00h). Heavy RAW in needles continued to decrease from 20 July, 18:00h until 22 July, 18:00h, which corresponded to rainy days with low PPFD. Then, from 24 July, trees responded to a series of sunny days with increased transpiration. Quite unexpectedly, heavy RAW reappeared strongly in needles on 25 July 18:00h, but by 28 July–6 August, the signal was completely lost.

Heavy RAW in roots showed larger short-term fluctuations than heavy RAW in needles. Concentration of heavy RAW in roots reached maximum at the end of heavy water administration (18 July, 18:00), then gradually decreased.

**Figure 3.**
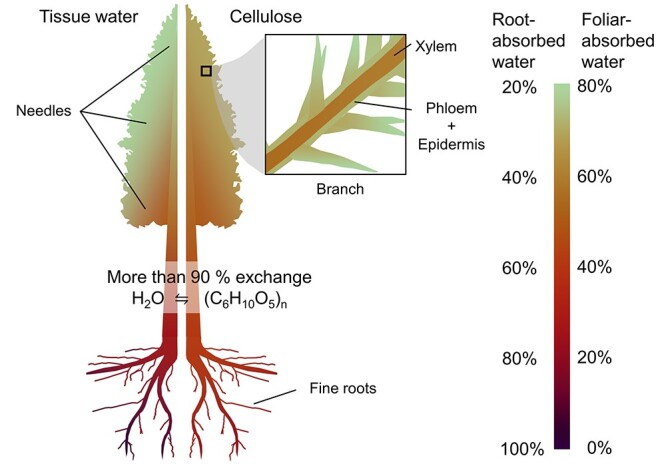
Origins of hydrogen and oxygen in tree biomass formed under rain conditions. During the first 3 days of the experiment, which were rainy, leaf water was mostly composed of FAW, and root water was mostly composed of RAW. Stem water was estimated to be around half FAW and half RAW (left side of the tree figure). FAW and RAW concentrations in local tissue were reflected in the hydrogen and oxygen of local biomass. More than 77% of hydrogen and oxygen in sugars should have exchanged with local water at the cambium. Thus, most of the hydrogen and oxygen incorporated into new leaf cellulose was derived from FAW, while that of new root cellulose was derived mostly from RAW, and that of new branch xylem was derived from roughly equal parts of FAW and RAW.

Very small but significant amounts of heavy FAW were detected in the root samples of some of the O/H and H/O trees (Figure S2 available as Supplementary data at *Tree Physiology* Online). The highest excess δ^18^O and δ*D* values observed in O/H and H/O roots were + 55.8‰ and + 464‰ on the VSMOW scale, respectively. Overall, the time-integrated peak area ratio of heavy FAW to RAW (Δ^18^O*_w_*/Δ*D*_w_ for O/H trees and Δ*D_w_*/Δ^18^O*_w_* for H/O trees in Figure 2) in tissue water across different organs, ranged from 4.8 in the needles to 0.02 in the roots when averaged over O/H and H/O trees (forming a gradient qualitatively expressed in the left side of Figure 3, see also Table 4). Pulse-labelling experiment by Augusti et al. (2006) reports similar gradient of deuterated water between leaves and stem xylem.

### Isotope ratios of drainage water

Less than half of the drainage water from the aboveground chambers originated from the heavy water sprayed on to needles (Table 2). About half of this drainage water was natural water originating from inside the trees and soil and a small fraction (4–8%) was heavy water administered to the ground chamber.

**Table 2:** Proportions of heavy water detected in drainage water

		O/H trees	H/O trees
Drainage water from upper chamber	Heavy hydrogen water fraction (τ*_H_*, %)	7.6 ± 2.0	41.5 ± 3.1
	Heavy oxygen water fraction (τ*_O_*, %)	47.9 ± 0.4	4.3 ± 0.2
	Natural water fraction (100 − τ*_H_* − τ*_O_*, %)	44.6 ± 2.0	54.2 ± 3.2
Drainage water from ground chamber	Heavy hydrogen water fraction (τ*_H_*, %)	35.6 ± 7.1	0.01 ± 0.00
	Heavy oxygen water fraction (τ*_O_*, %)	0.09 ± 0.02	28.3 ± 4.3
	Natural water fraction (100 − τ*_H_*–τ*_O_*, %)	64.4 ± 7.2	71.7 ± 4.3

O/H trees were labelled by spraying the needles with heavy oxygen water and watering the soil with heavy hydrogen water. H/O trees were labelled by spraying the needles with heavy hydrogen water and watering the soil with heavy oxygen water. The presence of natural water and both heavy waters in the upper chamber drainage water indicates bidirectional exchange between water on leaf/shoot surfaces and water inside the leaf.

About one–third of drainage water from the ground chambers consisted of heavy water administered to the soil, and the rest was natural water. The absence of heavy FAW (excess δ^18^O of O/H tree ground drainage water was 9.1‰, and excess δ*D* of H/O tree ground drainage water was 2.3‰) further indicates that no cross-chamber contamination of heavy water vapour occurred (Figure 1).

### Heavy FAW and RAW ratios in cellulose

Trends in the ratios of FAW to RAW assimilation into cellulose were examined through an analysis of heavy water percentage (τ) in α-cellulose (Table 3), where assimilation of both HDO + D_2_O and $ {\rm H}_{2}^{18}{\rm O}$ into cellulose was taken into account. ANOVA tests showed no significant difference between O/H and H/O treatments on heavy water percentage (τ) in α-cellulose extracted from needles, phloem + epidermis, xylem and roots (*P* = 0.40), nor a significant difference in τ between (i) needles and (ii) phloem + epidermis (*P* = 0.63). However, (i) needles, phloem and epidermis, (ii) xylem and (iii) roots were significantly different from each other (*P* = 1.8 × 10^−10^).

**Table 3:** Origins of hydrogen and oxygen in α-cellulose

	FAW, %	RAW, %	FAW/RAW
Average of O/H and H/O trees	(τ_Cell_/(τ_Cell_ + τ_Cell_))	(τ_Cell_/(τ_Cell_ + τ_Cell_))	(τ_Cell_/τ_Cell_)
Needles	69 ± 2	31 ± 4	2.2 ± 0.1
Phloem + epidermis	70 ± 4	30 ± 4	2.3 ± 0.2
Xylem	55 ± 4	45 ± 3	1.2 ± 0.1
Roots	20 ± 8	80 ± 8	0.2 ± 0.4
O/H trees	(τ_O_/(τ_O_ + τ_H_))	(τ_H_/(τ_O_ + τ_H_))	(τ_O_/τ_H_)
Needles	72 ± 2	28 ± 2	2.6 ± 0.1
Phloem + epidermis	74 ± 5	26 ± 5	2.9 ± 0.2
Xylem	58 ± 4	42 ± 4	1.4 ± 0.1
Roots	18 ± 7	82 ± 7	0.2 ± 0.4
H/O trees	(τ*_H_*/(τ*_O_* + τ*_H_*))	(τ*_O_*/(τ*_O_* + τ*_H_*))	(τ*_H_*/τ*_O_*)
Needles	67 ± 4	33 ± 4	2.1 ± 0.1
Phloem + epidermis	68 ± 5	32 ± 5	2.1 ± 0.2
Xylem	53 ± 3	47 ± 3	1.1 ± 0.1
Roots	24 ± 8	76 ± 8	0.3 ± 0.4

τ*_H_* is the percentage of hydrogen atoms in α-cellulose replaced by heavy hydrogen water. τ*_O_* is the percentage of oxygen atoms in α-cellulose replaced by heavy oxygen water. τ_Cell_ is weight proportion of (hydrogen + oxygen) in cellulose replaced by heavy water. Most of the hydrogen and oxygen incorporated into new leaf cellulose was derived from FAW, while that of new root cellulose was derived mostly from RAW, and that of new branch xylem was derived from roughly equal parts of FAW and RAW.

Average τ_Cell_ of FAW decreased in the order of (i) needles, phloem and epidermis, (ii) xylem and (iii) roots, decreasing with distance from shoots (Table 3). Similarly, average τ_Cell_ of RAW decreased in the order of (i) roots, (ii) xylem and (iii) needles, phloem and epidermis, decreasing with distance from roots. The ratio of assimilated FAW/RAW in α-cellulose ranged from a ratio of 2.2 in the needles to 0.2 in the roots (forming a gradient qualitatively expressed in the right side of Figure 3). It was estimated that about 93% of hydrogen and oxygen in fine root biomass went through exchange with root water (Table 4).

**Table 4:** Proportion of H and O exchange between root water and substrates of root biomass

		Root sampling date (month/day)								
Tree type	Variable	7/17 6:00h	7/17 18:00h	7/18 6:00h	7/18 18:00h	7/19 18:00h	7/20 18:00h	7/21 18:00h	7/22 18:00h	Average
O/H trees	***pq***_**ex**_	**0.99**	**0.97**	**0.95**	**0.93**	**0.95**	**0.92**	**0.87**	**0.86**	**0.93**
	*R*	0.14	0.09	0.16	0.26	0.26	0.30	0.35	0.31	0.24
	τ_Ht_	1.0	1.0	1.8	3.5	4.1	4.2	4.4	3.6	
	Δ*D*_wl_	0.0	0.4	0.9	1.7	2.9	3.3	3.1	2.7	
	Δ*D*_wt_	7.1	11.1	11.8	13.9	16.3	15.0	14.1	13.0	
	τ_Ot_	0.0	0.1	0.3	0.5	0.4	0.5	0.8	0.6	
	Δ^18^O_wl_	14.9	22.8	25.3	26.3	24.5	20.0	16.1	13.5	
	Δ^18^O_wt_	0.1	0.2	0.2	0.2	0.2	0.2	0.1	0.1	
H/O trees	***pq***_**ex**_	**0.96**	**0.95**	**0.93**	**0.95**	**0.95**	**0.90**	**0.91**	**0.90**	**0.93**
	*R*	0.19	0.17	0.23	0.24	0.41	0.59	0.54	0.44	0.35
	τ_Ht_	0.1	0.3	0.5	0.4	0.7	1.5	1.0	0.8	
	Δ*D*_wl_	22.0	31.5	33.8	33.7	29.1	23.2	18.6	15.6	
	Δ*D*_wt_	0.0	0.1	0.1	0.1	0.1	0.1	0.2	0.2	
	τ_Ot_	0.8	1.3	2.3	2.7	4.6	5.6	4.8	3.6	
	Δ^18^O_wl_	0.0	1.2	2.0	2.1	2.4	2.7	2.6	2.4	
	Δ^18^O_wt_	4.5	8.0	10.5	11.7	11.8	10.4	9.4	8.7	

Δ*D*_wl_, Δ*D*_wt_, Δ^18^O_wl_, Δ^18^O_wt_ of each date were calculated by integrating isotope values of leaf water and tissue water (root water) in Figure 2 over time, from the beginning of labelling (16 July, 18:00h) until the time of root sampling. Between 86 and 99% of the hydrogen and oxygen in translocated sugar was exchanged by those of local water before formation of root biomass. When needles were constantly wet (17–19 July), rate of exchange (*pq*_ex_) was higher than when needles were dry (20–22 July).

## Discussion

### Foliar and root water uptake

The first question to be addressed in this article concerns the rates of foliar water uptake and root water uptake and the process of absorption. In this study, even though water content increased with needle height position within saplings, the concentration of FAW varied little among upper, middle and lower needles (Figure S2 available as Supplementary data at *Tree Physiology* Online), so I will hereafter refer to the average trends of all needles (Figure 2). Up to 40% of needle water was replaced by FAW within the first 24 h of labelling. According to Berry et al. (2019)’s classification of leaves, the Japanese cedar needles in this study fall in the range of `high foliar water uptake rate' and `high water capacitance' (see Figure 4 in Berry et al. (2019)). Reports on FAW replacement of leaf water vary widely across species and experimental design, with other reports including 21% for a temperate conifer (*Araucaria angustifolia*) in overnight fog [see Figure 3a in Cassana et al.  (2016)], and nearly 100% in 3–8 h for *Pinus sylvestris* and *Fagus sylvatica* in fog (for other examples, see Lehmann et al. 2019).

**Figure 4.**
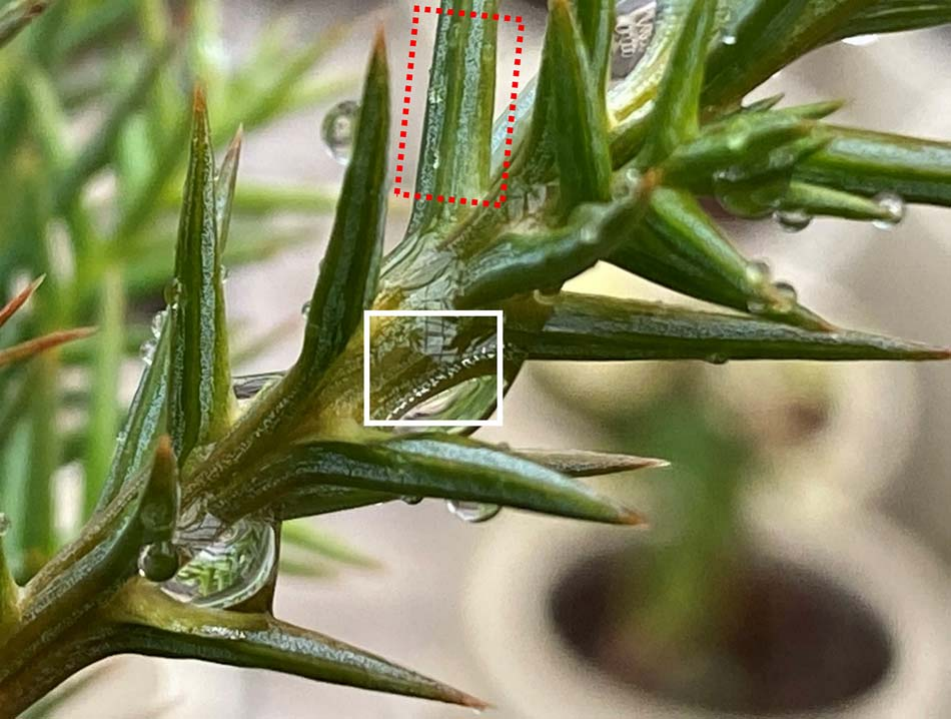
Droplets on *C. japonica* needles and shoots. Because of the low wettability of Japanese cedar needles, needles were mostly free from water droplets, and so stomatal bands (dotted red box) seemed to function unimpeded by water. Droplets tended to roll down needle surfaces to form larger drops with longer residence times at the surface of the shoot axis between needle bases (solid white box).

When rain falls on Japanese cedar, the water forms droplets that coalesce as they roll down and are trapped among the bases of needles at the shoot axis, keeping the needles mostly dry (Figure 4, Nanko et al. 2013). This is advantageous for CO_2_ uptake in a rainy climate (Hanba et al. 2004), but it limits foliar water uptake from needles and means that a significant portion of ‘foliar water uptake’ may happen via the uptake of liquid water through the epidermis of shoot axes instead of vapour through the stomata of needles (Mason Earles et al. 2016).

Because plants gain and lose water through bidirectional exchange at leaf surfaces (Farquhar and Cernusak 2005, Kim and Lee 2011, Goldsmith et al. 2017, Farquhar et al. 2021), the drainage water of the aboveground chambers in this experiment included not only run-off from spraying the needles but also water from inside the saplings (Table 2). Without further experiment, it would be hard to say how much heavy FAW from needle water was expelled through leaves back into the environment, but the 45–54% of drainage water composed of natural isotope ratios must have come either from natural water already inside the saplings at the start of labelling or natural water in the soil absorbed by the roots, conducted through the stem and transpired by the needles. The remaining 4–8% was root-absorbed heavy water, which was transpired in the same way (note that evaporation from the soil in the lower chamber cannot enter the upper chamber; Figure 1).

We can get a sense of the relative rates of foliar water uptake and root water uptake by noticing that the heavy RAW signals in roots and needles were initially smaller but more persistent than the heavy FAW signal in needles. Figure 2 and Figure S2 available as Supplementary data at *Tree Physiology* Online are dominated by the heavy FAW signals in needles which spiked dramatically during labelling, but disappeared within 4 days of the last labelled spray, while heavy RAW signals were more erratic and somewhat less pronounced, but still clearly present in roots more than 4 days, and in needles 7 days, after labelling. I deduce that the turnover of soil water took longer than the turnover of foliar-absorbed precipitation. In other words, heavy water uptake by shoots happened at a faster rate over a shorter period than heavy water uptake by roots, creating a lag between the heavy FAW and heavy RAW signal. This agrees with published research (Kabeya et al. 2007, Lehmann et al. 2018, 2019). Part of the lag can also be explained by fast bi-directional exchange of water through open stomata immediately after the pulse-labelling between saturated air within the chamber and the needles (Farquhar and Cernusak 2005, Helliker 2011, 2014, Goldsmith et al. 2017), and slow transport of water from roots to leaves (Volkmann et al. 2016, Ferrio et al. 2018).

The relative size of the isotopic signals of RAW versus FAW in needles can be explained by considering that the drainage water from the ground chambers contained 64–72% natural water (Table 2). Even allowing for bidirectional water exchange between roots and soil, I estimate that heavy water available to roots could not have exceeded averaged concentrations of much more than one-third during the labelling period because the concentrations of heavy water in the drainage water from the ground chamber was 28–36% and the maximum concentrations of RAW in fine roots was about 20% (Figure 2). The erratic nature of the heavy water signal may reflect temporal fluctuations of water flux direction in fine roots and erratic dispersion of water in the soil (Dawson 1993, Treydte et al. 2014) that would make labelling plants through soil water particularly tricky.

### Transport of FAW and RAW

The next step is to consider the transport of FAW and RAW within the saplings. The transport of RAW to needles is a direct prediction of the traditional model of transpiration (Philip 1966, Nobel 2009), but what about transport of FAW? Figure 2 shows that minima of RAW in needle water were accompanied by minima of transpiration, demonstrating that xylem flow changed direction depending on transpiration conditions (Eller et al. 2013, Goldsmith 2013).

Foliar-absorbed water signals in roots were close to zero but still significant in some of the O/H and H/O trees, as mentioned in section Isotopic signals of FAW and RAW in tissue water (Figure S2 available as Supplementary data at *Tree Physiology* Online). Cassana et al. (2016) report much larger concentrations of FAW in root water and possible release of FAW through the roots into the soil, creating FAW concentrations in soil + fine roots as high as 34%, but this only occurs for water-stressed trees (see Figure 3 in Cassana et al. (2016)). In my experiments, roots were watered only a few minutes after watering the shoots, most likely resulting in a bidirectional flow from leaves and roots towards stem, where water potential is lowest (as depicted in Figure 1b in Goldsmith (2013)). This also explains why drainage water from the ground chambers in this experiment contained only 0.01–0.09% FAW, much too small a signal to detect in the soil. Using spruce and fir saplings under moderate soil water conditions (volumetric water content above 25%), Berry et al. (2014) exposed needles of the saplings to deuterated water. Similar to our findings, they did not find FAW in the soil.

Nevertheless, the highest FAW in roots was detected on 17–21 July, and FAW concentrations in roots were highest for trees with the highest FAW concentrations in needles (trees C and D, Figure S2c and d available as Supplementary data at *Tree Physiology* Online). If we refer again to Cassana et al. (2016), we find that direction and volume of advection flow depend heavily on water potential, and that flow of xylem sap in the direction from leaves to roots is driven by an inverted gradient in the water potential across leaves, stem and roots. However, maximum FAW in roots observed in this study was 0.4–0.7% (Figure S2c and d available as Supplementary data at *Tree Physiology* Online), which is much smaller than 34% of FAW observed in Cassana et al. (2016). For example, the phloem to xylem flux ratio of water in popular is reported to be 0.19 at night (Windt et al. 2006). As Cassana et al. (2016) did not find significant amount of FAW in roots of well-watered trees, and flux of water in phloem is much smaller than in xylem, I believe that FAW in roots observed in this study was most likely transported to roots via phloem. In springtime, about 30% of gross primary production is translocated to roots via phloem (Epron et al. 2019), and both active phloem translocation and positive turgor in roots during my experiment are deduced because active fine root growth (up to 1 cm per day) was observed.

By this reasoning, opening the chambers to outside ventilation and thereby decreasing humidity on 19 July would lead to the normal water potential, increased transpiration, acropetal xylem sap flow and active water uptake by fine roots the following days. Indeed—except for 22 July, which was a dark day of 100% humidity (data not shown) with a minimum of transpiration—RAW in the needles was observed through 25 July. Absence of RAW in needles on 22 July signifies that sap had been flowing in the inverted, basipetal direction. By then, the trees had been receiving only tap water for 3 days, and any heavy FAW still in the needles would have been lost by transpiration or diluted by newly absorbed tap water. However, some of the heavy FAW could still have existed in the phloem of branches and stems, which have capacitance and can store some amount of water (Sevanto et al. 2011, Steppe et al. 2018). I speculate that the combination of high humidity, needle wetting and minimum transpiration triggered inversion of the water potential gradient and caused heavy FAW in the needles to flow down to branches or stems through xylem, and a small but persistent supply of FAW to the roots through phloem until 22 July.

Conditions on both 24 and 25 July were sunny and dry, and, by the above reasoning, active transpiration would have returned the xylem sap flow to the familiar, roots-to-needles direction, replacing tap water in needles with heavy RAW transported from the branch, stem, roots and soil. Heavy RAW in needles reached the second maximum on 25 July at 18:00h, but all signals in root and needle water were lost by 6 August, probably due to repeated watering with tap water during and after transplanting and active transpiration under drier weather.

### Assimilation of FAW and RAW into cellulose

Supporting my hypothesis, isotope analysis of the biomass formed during the rainy season, where a rain event was emulated by the dual-labelling experiment, revealed that a significant percentage of hydrogen and oxygen in the cellulose was derived from FAW (Table 3). One early study of hydrogen and oxygen isotopes in plant biomass discusses water absorbed by roots as a source of hydrogen and oxygen but does not explicitly mention water absorbed by leaves (Sternberg 1989). Hydrogen and oxygen contribution from atmospheric vapour to hydrogen and oxygen isotopes of tree rings were accounted for by the current models (Roden et al. 2000, Farquhar and Cernusak 2005, Studer et al. 2015), however, they do not account for contributions from liquid FAW. I suggest that, textbook figures explaining water use in photosynthesis (e.g., see Box Figure 5.2 in Lambers and Oliveira 2019) are inadequate, because most of tree’s radial growth happens during and immediately after rain events (Zweifel et al. 2021), and we therefore need new figures that include foliar uptake of liquid water, at least for trees growing under climates with wet growing seasons (such as Figure 3). Furthermore, we have discussed evidence that water potential drives transport of FAW and RAW to set up a FAW/RAW gradient in tissue water, which is supported by previous labelling experiment with deuterated water (Augusti et al. 2006). I propose that this FAW/RAW gradient in tissue water is then embedded in the cellulose (as illustrated in Figure 3), because newly formed biomass, such as needles, wood and roots, carries time-integrated FAW/RAW signature of local water at the cambium (Roden et al. 2000, Barnard et al. 2007, Gessler et al. 2007).

Let us combine this study’s results with current knowledge to examine how this process might play out. First, as discussed under Transport of FAW and RAW, we assume that the uptake and transport of FAW and RAW create a gradient in the tissue water that can shift according to water status. Photosynthesis assimilates available hydrogen and oxygen from leaf water (and carbon from CO_2_) and embeds the leaf water signal of FAW/RAW into carbohydrates (DeNiro and Epstein 1979, Farquhar et al. 1998). These carbohydrates may be used locally or translocated to other parts of the plant. Although we do not know if and at what rate the carbohydrates exchange oxygen (and hydrogen) with local water during phloem loading, transport and unloading (Gessler et al. 2014), even with lack of direct data, we expect carbohydrates dominated by sucrose to carry isotopic signals acquired in production to other parts of the plant because sucrose has no free carbonyl group to exchange oxygen atoms with local water. However, various authors have found that there is a significant rate of exchange during synthesis of cellulose (Hill et al. 1995, Cernusak et al. 2005, Sternberg 2009), which would encode the local ratio of FAW/RAW into the cellulose.

In leaves, the isotopic signals of biomass should be a function of the integrated isotopic signals of leaf water (Barnard et al. 2007, Gessler et al. 2007), and about half of leaf water can be replaced with FAW during a rain event (Figure 2, Figure S2 available as Supplementary data at *Tree Physiology* Online). Away from the site of photosynthesis, we can expect exchange during cellulose formation to shift the isotopic signals of cellulose from FAW towards RAW as we move in the direction from leaves to roots. Therefore, the signal in tree rings should be a function of the FAW/RAW signal in cambial water and depend on the average fractions of carbon-bound hydrogen available for exchange (*q*_ex_) and oxygen available for exchange (*p*_ex_). In this study, the similarity of trends of heavy water distributions in tissues (Figure 2 and Figure S2 available as Supplementary data at *Tree Physiology* Online) and signals in α-cellulose (Table 3) between the O/H and H/O trees supported the assumption that *q_ex_* ≈ *p_ex_* in our approach. The signal in roots should be a function dominated by the RAW signal.

The substitution of τ_Ht_ and τ_Ot_ with isotope ratios of bulk root biomass (which includes cellulose and other organic substance) sampled from 16 July to 25 July gave averaged *pq*_ex_ values of 0.93 for both the O/H trees and the H/O trees (Table 4), and samples of α-cellulose extracted from roots yielded *pq*_ex_ = 0.82 for the O/H trees and *pq*_ex_ = 0.73 for the H/O trees (calculated from data in Tables 3 and 4 using Eq. (16)). Values of *pq*_ex_ were especially high when needles were constantly wet (17–19 July), supporting our hypothesis that higher water potential causes increased exchange of hydrogen and oxygen between sugars and local water at cambium. As time elapsed from the start of labelling, a gradual increase in *r* was also observed, indicating deposition of deuterium and oxygen-18 into newly formed root biomass.

Among the reported *pq*_ex_ values, these are on the high end, but not unreasonably so. For various species and growing conditions, other authors have found that, *q*_ex_ during biomass synthesis to be close to 0.40 (Yakir and Deniro 1990, Terwilliger and Deniro 1995, Roden and Ehleringer 1999, Waterhouse et al. 2002) and *p*_ex_ between 0.38 and 0.77 (see Table 9 in Cernusak et al. 2005). *p*_ex_ is known to show seasonal variations between spring and summer, with the highest exchange observed at the beginning of the growing season (*p*_ex_ = 0.76 for *Fagus sylvatica*, Offermann et al. 2011), when elevated temperatures are known to promote enzymatic conversion of starch to sugar to supply energy for new cell wall formation (Begum et al. 2010).

Since the trees on my study were well watered, one potential explanation for high *p*_ex_ (and *q*_ex_) could be that foliar water uptake caused leaf water and the carbohydrates contained within to be transported to the stem and roots via phloem (Laur and Hacke 2014*a*, Schreel et al. 2020), thereby increasing the sugar content in phloem and parenchyma cells—and hence turnover time of carbohydrate pool—at the branch, stem and roots. As cells at the cambial zone absorb water and expand, solutes in their cytoplasm are diluted. To maintain constant osmotic pressure in the cytoplasm, expanding cells actively break down sucrose into glucose and fructose (Koch 2004), which may explain the unusually high exchange of oxygen (and hydrogen) between sugars and local water observed when needles were constantly wet (Table 4).

Eventually, the labelling signal of water inside trees would be either incorporated into the structural components, such as tree-ring cellulose, or released back into the environment. Although significant labelling signals in organic matter were found in new roots that were sampled during the labelling period (Tables 3 and 4), replanting the trees on July 26 also allowed me to later isolate roots formed in the post-labelling period, when there would have been less, if any, labelled primary photosynthate available for cellulose production. When samples were taken in January 2020, there was a possibility that, compared with samples from July 2019, samples from January 2020 may contain lesser label because biomass formed during the rest of the growing season dilutes the labelling signal. However, dilution of the label by the non-labelled biomass was not so large, presumably because growth slows down after the end of the rainy season (Kagawa et al. 2005), and so—except for the roots, which would be formed from stored labelled carbohydrates—hydrogen and oxygen isotope ratios of needle, phloem + epidermis and wood cellulose extracted in January showed much higher isotope ratios than natural abundance. If the post-labelling roots sampled in January should contain any signals from heavy water supplied in July, it should come from stored carbohydrates, however significant labelling signals were not found in any of the thick, post-labelling roots (diameter up to 3 mm) sampled from all six labelled trees (*P* = 0.095, Table S2 available as Supplementary data at *Tree Physiology* Online). In a previous study, my laboratory successfully detected signals from photosynthate labelled with ^13^CO_2_ in July in roots formed later that year (Kagawa et al. 2006*b*). Since no such D and ^18^O signals were found in the present study, I deduce a complete exchange of D and ^18^O signals in the carbohydrate pool with medium water and/or fast turnover of the pulse-labelled carbohydrate pool within the 6 months between labelling in July and sampling the following January. Since 93% of hydrogen and oxygen in substrates for roots on average was replaced with root water within a week (Table 4), hydrogen and oxygen of the carbohydrate pool might have been turning over at a much faster rate than carbon. In the coming years, I plan to analyse hydrogen and oxygen isotope ratios of tree rings formed in years following labelling to see if hydrogen and oxygen isotope signals also appear in later rings, as ^13^C signals appear in later rings after ^13^CO_2_ labelling (Kagawa et al. 2006*a*).

Current mechanistic models explaining tree-ring hydrogen and oxygen isotope ratios are based mainly on experiments in which trees were grown in hydroponic facilities without leaf wetting (Roden et al. 2000). However, in the present study, more than half of the hydrogen and oxygen in the cellulose formed during rainy season in the current-year’s branch wood was derived from FAW, so this practice is inadequate for explaining tree-ring hydrogen and oxygen isotopes from humid areas.

In order to discuss hydrogen and oxygen allocation patterns of trees, we can make a comparison to carbon allocation patterns of trees, which can be explained by a source–sink model (Lacointe 2000). There is only one source of carbon for autotrophic trees: leaves (to be more exact, photosynthetic organs). However, there are at least two sources of hydrogen and oxygen: FAW (in vapour and liquid form) and RAW. According to source–sink models of carbon allocation, each non-green organ (sink) draws carbon from the nearest source with the shortest distance. Similarly, in this study, cellulose of leaves contained mostly FAW, that of roots contained mostly RAW, and that of branch wood contained roughly equal parts of FAW and RAW (Table 3), thus suggesting that this rule also holds true for hydrogen and oxygen allocation of trees. As foliar uptake of liquid water was accompanied by an increased exchange of hydrogen and oxygen (up to 99%) between local water and carbohydrate pool (Table 4), I conjecture that, during a rain event following days of sunshine, hydrogen and oxygen isotopic information in sugars, which carries leaf evaporative enrichment signals (elevated δ*D* and δ^18^O) from the time of photosynthesis, may be mostly overwritten by local water at the cambium. If we take into account that the most of stem radial growth happens during and immediately after rain events (Zweifel et al. 2021), and growth reaches maximum speeds during this period (Kagawa et al. 2005, Rossi et al. 2006, Zweifel et al. 2021), foliar water uptake during and immediately after rain events is expected to make a significant contribution to tree-ring hydrogen and oxygen isotopes in monsoon Asia. In a natural setting, oxygen isotope ratios of absorbed rainwater will not be too different from those of soil water (Treydte et al. 2014), and therefore FAW/RAW changes of tissue water under rain conditions will not directly affect oxygen isotope ratios of plant biomass in a significant way. However, during leaf-wetting events, increased oxygen exchange between local cambial water and sugars was observed (*p_ex_*, which is normally about 0.4, has increased to 0.93). This means 93% of evaporative enrichment signals (high δ^18^O) contained in leaf sugar can be overwritten with low δ^18^O signals from local cambial water, which may result in lower δ^18^O values of tissues formed under wet conditions than the current mechanistic model predicts.

Results of this study also suggested a close connection between foliar water uptake and canopy interception. The greatest foliar water uptake was observed immediately after I started wetting the needles (Figure 2, Figure S2 available as Supplementary data at *Tree Physiology* Online). The greatest canopy interception is reported to happen at the onset of a rain event, when needles start to get wet (Iida et al. 2017), and the results of this study suggested foliar water uptake as one of the key mechanisms driving active canopy interception at the beginning (within 12–24 h after the onset) of a rain event. Among forests in the world, coniferous forests with closed canopy shows the highest canopy interception losses, up to 48% of total rainfall (Hörmann et al. 1996).

Even when rain is falling in a forest, the air within a canopy is not saturated (Murakami 2006, Dunkerley 2009), suggesting the possibility of canopy-air humidity decrease caused by net foliar ‘vapour’ uptake. Some studies demonstrate foliar vapour uptake under high humidity conditions by up-regulation of aquaporin (Laur and Hacke 2014*a*, 2014*b*) or through deliquescence of salt (Wang et al. 2016, Coopman et al. 2021). Even in the absence of physical leaf wetting, net foliar vapour uptake may happen, when water vapour concentrations in leaf intercellular gas spaces are less than those in the atmosphere (Vesala et al. 2017, Cernusak et al. 2018). In the future, dual-isotope labelling method developed in this study could be used to further investigate these processes.

## Conclusions

As far as I know, this is the first study that quantifies relative contributions of FAW and RAW to the biomass of terrestrial plants. I found that foliar water uptake reached equilibrium within 12–24 h in *C. japonica* saplings, and the ratio of FAW to RAW increased from roots to branches to leaves, creating isotopic gradients that were embedded in cellulose. More than half of the hydrogen and oxygen in new branch wood originated from FAW in liquid or vapour form. Although these results may be the most representative of trees with rainy growing seasons, they clearly demonstrate the need to overhaul the common misconception among non-specialists that hydrogen and oxygen in plant biomass are overwhelmingly supplied by soil water. The results of these experiments cannot be simply extrapolated to mature trees, because mature trees may rely more on stored carbohydrates remobilized from ray parenchyma cells, and water stored in heartwood may move radially to cambium through ray parenchyma (Nakada et al. 2019, Treydte et al. 2021), so that hydrogen and oxygen from these sources can be eventually incorporated into biomass. Future research on mature trees under field conditions over a longer time period is required to determine exactly how much hydrogen and oxygen in plant biomass originates from FAW.

The dual-isotope pulse-labelling method can be used to further explore this topic and other biological processes related to this study, such as absorption and transport processes of FAW, and clarification of whether FAW is transported via xylem, phloem or some combination of the two. We might also address how much FAW is used for production of molecular oxygen through photosynthesis and how much exchange happens between FAW and oxygen of atmospheric CO_2_ through stomatal exchange. In the future, experiments with stable isotopes, if conducted in a closed system with closely monitored isotopic mass balances between plants and the surrounding environment, may even enable us to quantify the absolute net foliar water uptake into plants.

The dual labelling method with HDO + D_2_O and $ {\rm H}_{2}^{18}{\rm O}$ first implemented in this study offers a new tool to study the uptake, transport and assimilation processes of water in terrestrial plants, and successfully enabled separate and simultaneous labelling of FAW and RAW. Using this method, I found conclusive evidence that FAW can be a significant source of hydrogen and oxygen in plant biomass, such as tree rings, formed during rainy season.

## Conflict of interest

None declared.

## Authors’ contributions

A.K. has done all the work.

## Data and materials availability

All the data and materials supporting the findings of this study are available from the corresponding author upon reasonable request.

## Supplementary Material

Supplementary_file_tpac055
