# Supplementary material for: Foliar water uptake as a source of hydrogen and oxygen in plant biomass
Source: Tree Physiol. 2022 May 11;42(11):2153–73. doi: 10.1093/treephys/tpac055 (PMC9652008; doi:10.1093/treephys/tpac055)
Supplement: Supplementary_file_tpac055 [file supplementary_file_tpac055.docx]

**Supplementary data for:**

Foliar water uptake as a source of hydrogen and oxygen in plant biomass

Akira Kagawa

Wood Anatomy and Quality Laboratory, Forestry and Forest Products Research Institute, Tsukuba, 3058687 Japan.

**Table S1 Hours of solar radiation and amounts of transpiration.**

|  | Radiation hours  (h) | Transpiration  (g) |
| --- | --- | --- |
| 20 July | 9.6 | 34.5 ± 8.8 |
| 21 July | 6.1 | 18.6 ± 5.7 |
| 22 July | 1.7 | 3.0 ± 2.0 |
| 24 July | 10.4 | 39.6 ± 8.4 |
| 26 July | 11.6 | 52.5 ± 14.1 |

Transpiration amounts of tree A-F were measured by weighing each pot before and after watering. Transpiration was proportional to radiation hours, with trees transpiring an average of 53 g each on a sunny day and between 3 and 35 g each on rainy/cloudy days.

**Table S2 Isotope ratios of α-cellulose extracted from roots sampled in January.**

|  | δD (‰ VSMOW) | δ^18^O (‰ VSMOW) |
| --- | --- | --- |
| O/H trees | -65.7 ± 3.4 | 34.0 ± 0.1 |
| H/O trees | -42.5 ± 3.8 | 35.3 ± 0.3 |
| Control trees | -10.2 ± 4.0 | 33.8 ± 0.5 |

Roots for this analysis were sampled in January, 2020, approximately six months after labelling. No significant increase of D and ^18^O signals were found, indicating a complete exchange of D and ^18^O in the carbohydrate pool with medium water and/or complete turnover of the carbohydrate pool within the six months


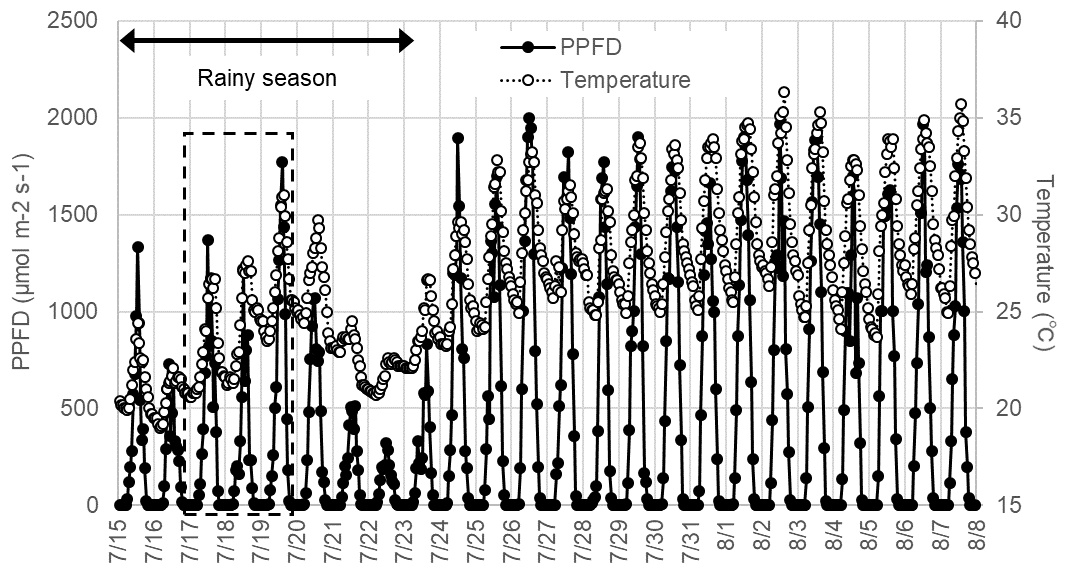


Figure S1 Hourly temperature and PPFD data. Labelling with heavy water was conducted from 16 July at 18:00 to 19 July at 18:00 (dashed box). Photosynthetic photon flux density (PPFD) was estimated from radiation data.


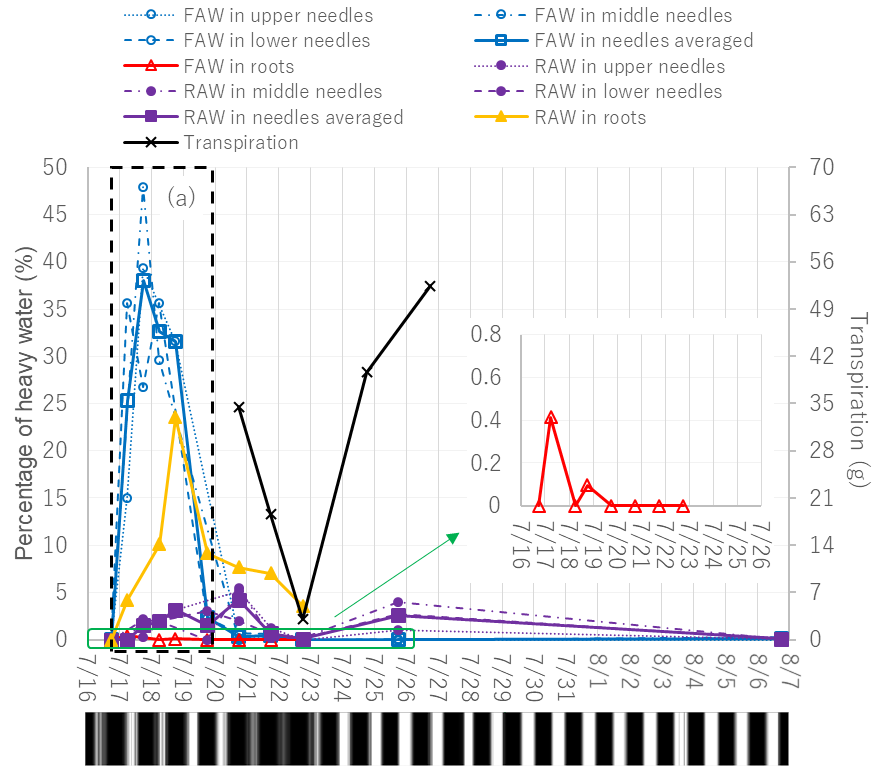


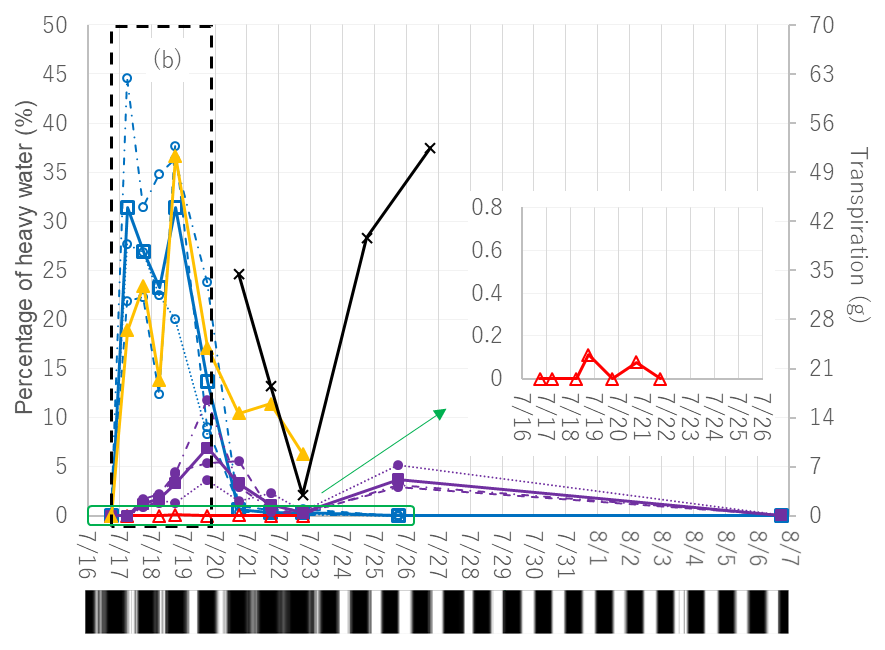


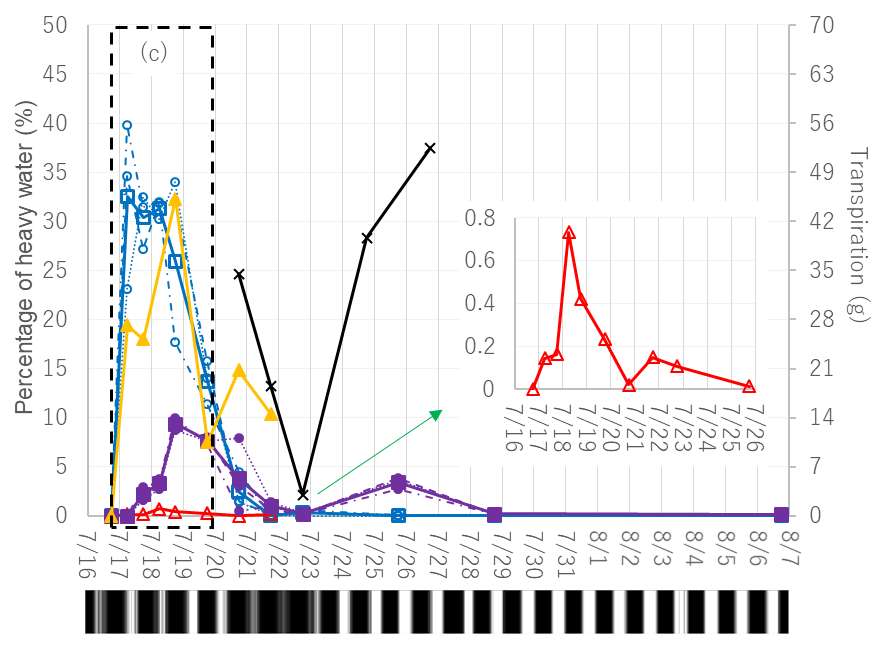


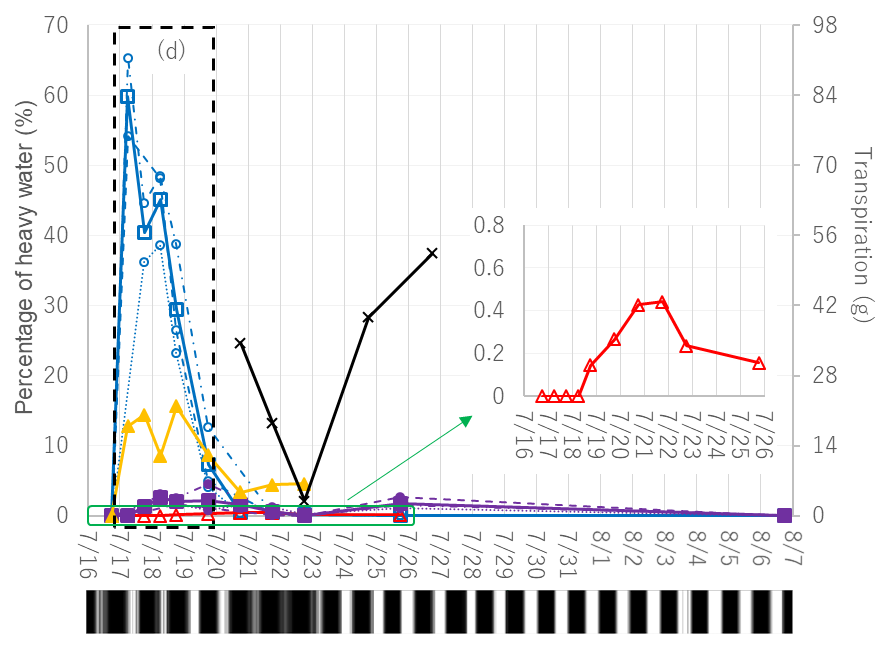


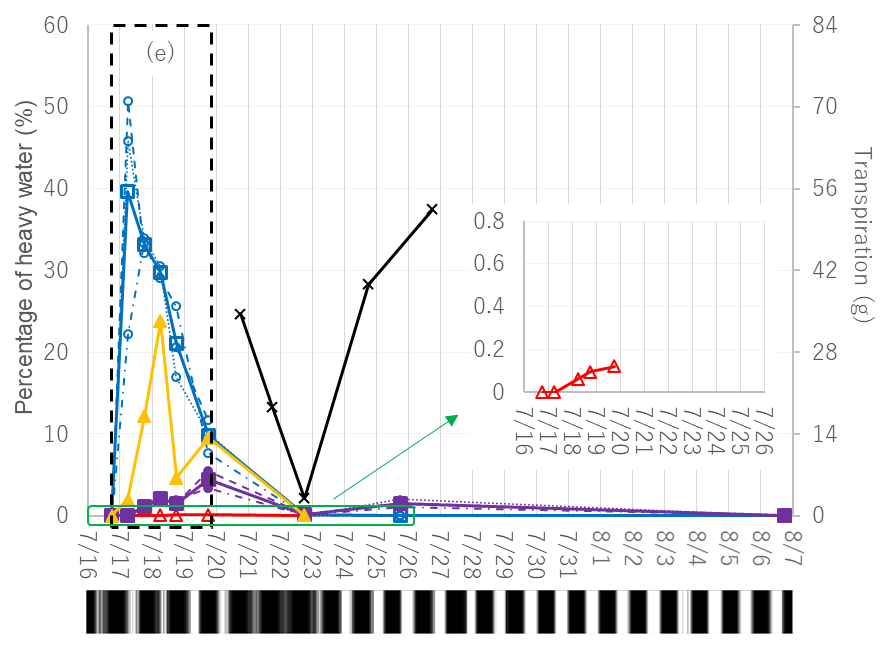


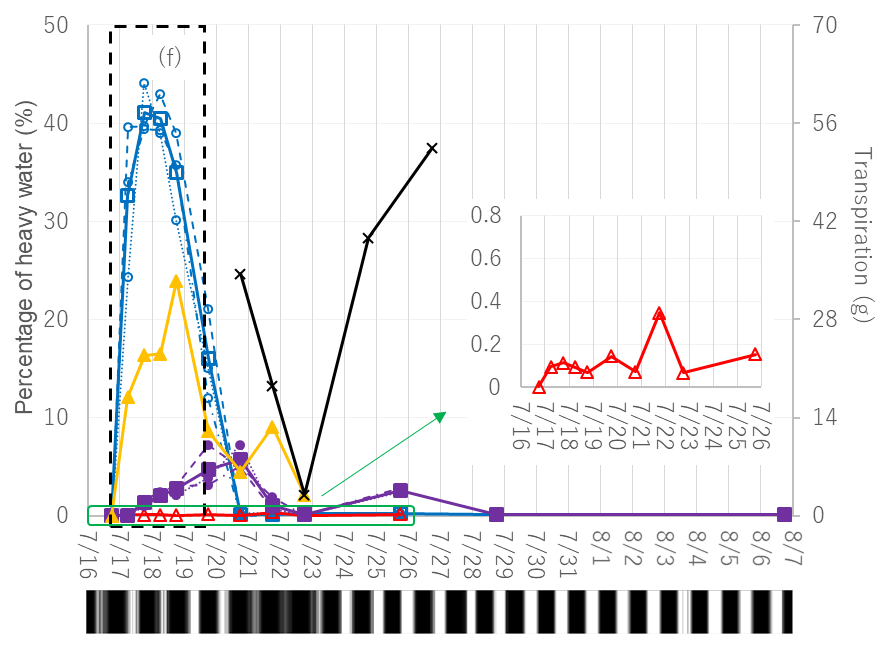


Figure S2 Absorption and transport of heavy foliar-absorbed water (FAW) and heavy root-absorbed water (RAW) within trees. (a) Percentages of applied heavy water in needles and roots of tree A, (b) tree B, (c) tree C, (d) tree D, (e) tree E, and (f) tree F. Labelling with heavy water was conducted from 16 July 18:00 to 19 July at 18:00 (dashed box). Inset in each subfigure shows percentages of heavy foliar-absorbed water in roots.
